# Dispersal and oviposition patterns of *Lycorma delicatula* (Hemiptera: Fulgoridae) during the oviposition period in *Ailanthus altissima* (Simaroubaceae)

**DOI:** 10.1038/s41598-022-14264-0

**Published:** 2022-06-15

**Authors:** Minhyung Jung, Jung-Wook Kho, Do-Hun Gook, Young Su Lee, Doo-Hyung Lee

**Affiliations:** 1 Department of Life Sciences, Gachon University, Seongnam-si, Gyeonggi-do 13120 South Korea; 2 Gyeonggi Agricultural Research and Extension Services, Hwaseong-si, Gyeonggi-do 18388 South Korea

**Keywords:** Behavioural ecology, Entomology

## Abstract

The spotted lanternfly (SLF), *Lycorma delicatula* (Hemiptera: Fulgoridae), has the potential to become a global pest and is currently expanding its range in the United States. In this study, we investigated the dispersal patterns of SLF in *Ailanthus altissima* during its oviposition period in South Korea using a fluorescent marking system. Oviposition patterns of SLF were then analyzed by surveying egg masses in *A*.* altissima* patches. The recapture rate of fluorescent-marked SLF rapidly decreased to 30% within the first two weeks. During the oviposition period, seven cases of among-patch dispersal of SLF adults were observed. The minimum distance that SLF could have traveled to achieve these among-patch dispersal events ranged from 10 to 1740 m, with most events spanning under 60 m. Also, the number of *A*.* altissima* trees on which fluorescent marked SLF were detected increased until September. Based on the egg mass survey, a total of 159 egg masses were detected from 38 out of 247 *A*.* altissima* trees. Furthermore, 79.2% of egg masses were located < 2.5 m above the ground. Finally, a generalized linear mixed model showed that tree height and diameter at root collar (DRC) of *A*.* altissima* trees had significant effects on the number of egg masses.

## Introduction

*Lycorma delicatula* (White) (Hemiptera: Fulgoridae), spotted lanternfly (SLF), is an invasive agricultural pest native to China^1,2^. This pest has invaded South Korea, Japan, and the United States, causing serious problems in agricultural and forest landscapes^1,3–6^. In South Korea, SLF was first reported in 2004, and the area of infestation increased over 8000-fold from 2006 to 2010^7–10^. In the US, it was first detected in Berks County, Pennsylvania in 2014. Since then, SLF infestation has expanded to nine different states including New Jersey, Virginia, and Delaware by 2022^3,6,11^. Furthermore, recent modeling studies predicted that SLF may become a global threat. Portions of Asia, Oceania, South America, North America, Africa and Europe might be susceptible to its invasion based on their temperature profiles^1,6^.

SLF has a broad range of host plants which facilitate the successful establishment of this pest in new areas. It is known to feed on more than 70 plant species including various ornamental and fruit trees such as apples (*Malus* spp.) (Rosaceae), grapes (*Vitis vinifera*) (Vitaceae), and peaches (*Prunus persica*) (Rosaceae), as well as other woody trees such as black locust (*Robinia pseudoacacia*) (Fabaceae), willow tree (*Salix* spp.) (Salicaceae), and Korean evodia tree (*Evodia danielli*) (Rutaceae)^4,10^. Moreover, in invaded areas including South Korea and the US, SLF is known to switch hosts during its development, with the range of host plants becoming narrower through its univoltine life cycle^10^. In temperate region, nymphs feed on a variety of host plants from 81 taxa, after emerging from eggs in May^12–15^. As they start to develop into adults from late July, SLF adults feed on a narrower range of host plants of 47 taxa. Finally, the adults oviposit egg masses on hosts belonging to 28 taxa as well as non-plant materials such as stones and metal fences between late September and November^4,10,12,13^.

Among various host plants of SLF*, Ailanthus altissima* (Simaroubaceae), the tree-of-heaven, is native to Southeast Asia and known as one of the major host plants in China, South Korea, and the US^4,16^. Furthermore, *A*.* altissima* has already been introduced and established in multiple continents including East Asia, Europe, and North America, making it readily available for SLF even in areas where the insects have yet to invade^17^. Previous studies indicate the importance of *A*.* altissima* as a major host plant of SLF. First, *A*.* altissima* is known as the most preferred and suitable feeding host of SLF along with *V*.* vinifera*. Lee et al.^16^ observed a high survivorship of SLF nymphs and adults on *A*.* altissima* as well as their preference for this plant compared with different ornamental and fruit trees. Second, *A*.* altissima* was reported to be one of the most preferred oviposition sites^4,10^. Liu et al.^18^ found that *A*.* altissima* was one of the four oviposition host plants favored by this insect along with black cherry, black birch, and sweet cherry in Pennsylvania, US. Finally, *A*.* altissima* contains high concentrations of cytotoxic alkaloids, which SLF may utilize for their own defence against predators^4,13,19^.

Therefore, it is essential to understand the abundance and distribution of SLF on *A*.* altissima* in order to develop effective management programs. Previous studies demonstrated that SLF adults shift host plants from woody and non-herbaceous plants, on which the insects feed as they emerge in July, towards *A*.* altissima* between September and November^12,13,18^. In addition, SLF adults are known to mate and reproduce during this period utilizing multiple substrates including *A*.* altissima* for oviposition^20^. However, the dispersal patterns of SLF adults within and among *A*.* altissima* patches after their arrival have not yet been investigated. Especially, given that SLF is a univoltine species in the invaded areas, investigating the dispersal patterns of this pest on *A*.* altissima* and characterizing their oviposition pattern can provide valuable information for its management.

In our study, we surveyed the abundance and distribution of SLF on *A*.* altissima* patches from September to November when SLF adults are known to shift their host plant to *A*.* altissima* and lay eggs^13,18^. In particular, to investigate the dispersal pattern of SLF within and among different *A*.* altissima* patches, we tracked the movement of SLF using a fluorescent marking system (FMS)^21,22^. Then, in December, we surveyed the location, number, and size of SLF egg masses on *A*.* altissima* trees and analyzed the oviposition patterns relative to the traits of *A*.* altissima* surveyed.

## Results

### Effect of fluorescent marking on SLF

**Figure 1:**
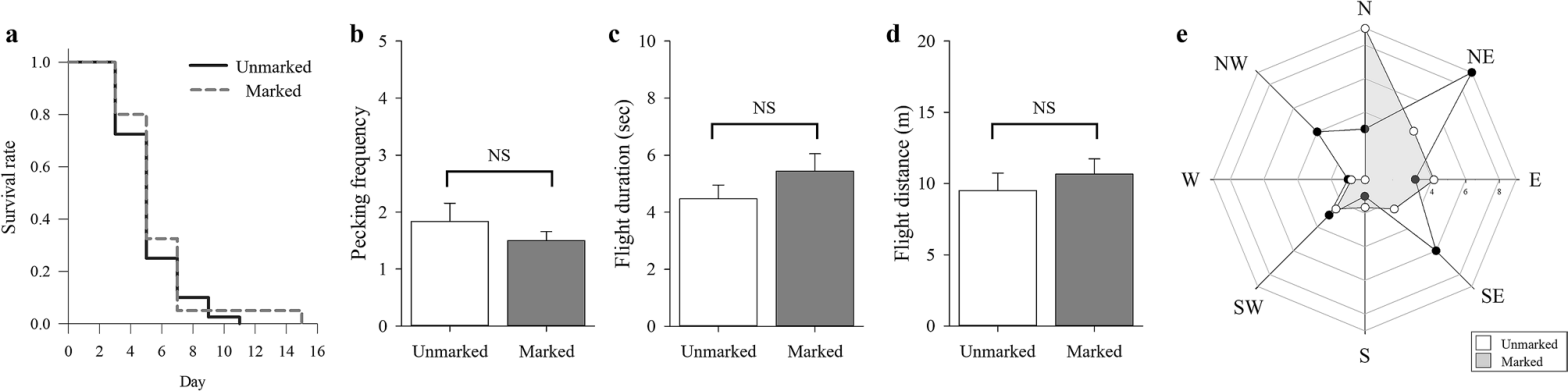
Assessment of the effect of fluorescent marking on performance of *Lycorma delicatula*. (**a**) survival rate of fluorescent-marked and unmarked *L*.* delicatula* over 16 days, (**b**) number of pecks to initiate the flight of *L*.* delicatula*, (**c**) flight duration, (**d**) flight distance, and (**e**) flight direction. In (**e**), points’ distance from the center indicates the number of trials in which individuals flew in a given direction. NS indicates no significant difference detected between two groups.

Fluorescent marking did not significantly affect the survivorship and flight behavior of SLF adults. First, the survivorship of SLF was not significantly affected by fluorescent marking (χ^2^ = 0.81, *df* = 1, *P* = 0.37; Fig. 1a). Second, no significant effect of fluorescent marking on flight behavior of SLF was observed. Fluorescent-marked and unmarked control groups did not show significant differences in the number of pecks on the forewings required to initiate flight (*t* = 0.93, *df* = 1, *P* = 0.36; Fig. 1b), flight duration (*t* = 0.71, *df* = 1, *P* = 0.48; Fig. 1c), and flight distance (*t* = 1.23, *df* = 1, *P* = 0.22; Fig. 1d). Moreover, the overall flight direction was not substantially different between fluorescent-marked and unmarked groups; the flight direction of SLF generally aligned along the north and northeast axes (Fig. 1e).

### Dispersal pattern of SLF on *A*.* altissima*

**Figure 2:**
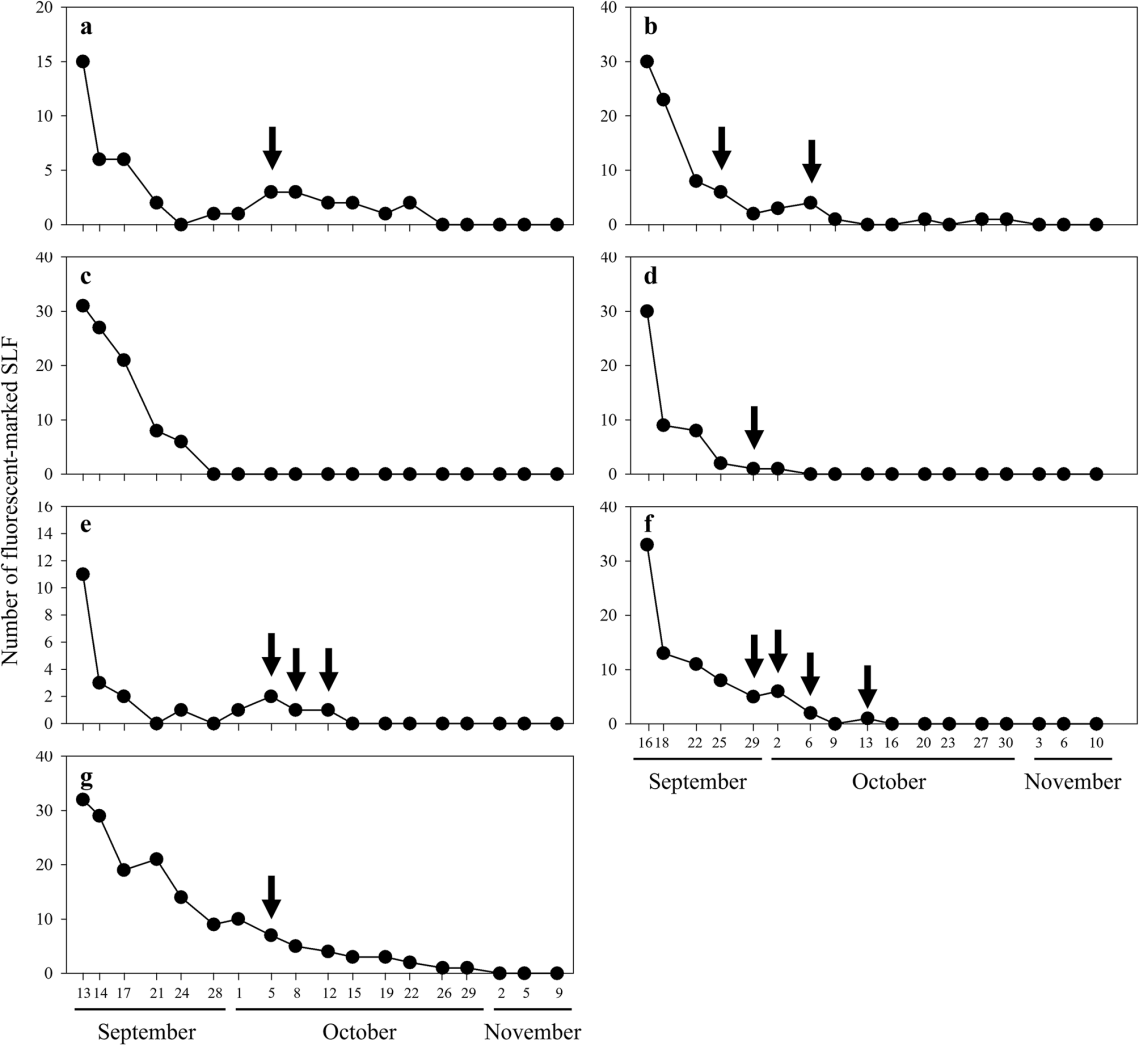
Number of fluorescent-marked *Lycorma delicatula* observed from *Ailanthus altissima* patches from September to November among patches A–D (**a**, **c**, **e**, and **g**) in Tan stream, and patches E–G (**b**, **d** and **f**) in Gyeongan stream. Black arrow indicates the date on which *L*.* delicatula* that had been marked from different patch at the onset of the experiment was observed.

The number of fluorescent-marked SLF adults showed a large decrease during the first two weeks of survey yielding average recaptures rates of 8.7 ± 6.6% (Mean ± SE) and 10.5 ± 4.3% in Tan stream and Gyeongan stream, respectively (Fig. 2). After the first two weeks, fluorescent-marked SLF adults were occasionally detected from both streams until late October, with no fluorescent-marked individuals observed in November.

**Figure 3:**
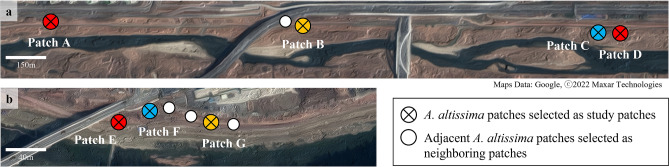
*Ailanthus altissima* patches selected to study dispersal and oviposition patterns of *Lycorma delicatula* in (**a**) Tan stream and (**b**) Gyeongan stream. Colors filled in each patch indicates the color of fluorescent paint for marking of *L*.* delicatula* adults. See “Materials and methods”for descriptions of study and neighboring patches.

A total of 12 events were recorded in which SLF individuals marked from different patches at the onset of the experiment were observed (Fig. 2; Table 1). Because fluorescent marking in this study did not allow us to identify different individuals, we were able to confirm seven among-patch dispersals among the 12 events recorded (Table 1). For example, it cannot be confirmed whether an SLF individual observed in patch E on October 6th was a new individual that had moved from patch F or the same individual observed on September 25th that remained in patch E. For this reason, we assigned ‘unidentified’ for among-patch dispersal with no record of minimum distance moved in Table 1. Minimum dispersal distance was estimated for the seven confirmed among-patch dispersal cases by measuring the shortest distance between two patches where SLF was marked and marked SLF was observed. Among-patch dispersal was recorded in all study patches except for patch B (Fig. 2; Table 1). In addition, we observed fluorescent-marked SLF in neighboring patches adjacent to study patches. In Tan stream, a fluorescent-marked SLF from patch B was detected two times in the neighboring patch on September 28th and October 1st (Fig. 3). In Gyeongan stream, fluorescent-marked SLF from patch E were observed three times from a neighboring patch located between patch F and G, on September 22nd, 25th, and 29th, and another fluorescent-marked individual from patch G was detected on September 29th (Fig. 3).

**Table 1:** Characteristics of fluorescent-marked *Lycorma delicatula* adults observed from different study patches in Tan and Gyeongan streams.

Site	Date	Original patch^a^	Observed patch	No. of *L*.* delicatula* observed	Among-patch dispersal	Minimum distance moved^c^
Tan stream	Oct 5th	Patch C	Patch A	1	Confirmed	1740 m
	Oct 5th	Patch A/Patch D	Patch C	1	Confirmed	1740 m/10 m
	Oct 8th	Patch A/Patch D	Patch C	1	Unidentified	–
	Oct 12th	Patch A/Patch D	Patch C	1	Unidentified	–
	Oct 5th	Patch C	Patch D	1	Confirmed	10 m
Gyeongan stream	Sep 25th	Patch F	Patch E	1	Confirmed	20 m
	Oct 6th	Patch F	Patch E	1	Unidentified	–
	Sep 29th	Patch E	Patch F	1	Confirmed	20 m
	Sep 29th	Patch F	Patch G	1	Confirmed	60 m
	Oct 2nd	Patch F	Patch G	2	Confirmed^b^	60 m
	Oct 6th	Patch F	Patch G	1	Unidentified	–
	Oct 13th	Patch F	Patch G	1	Unidentified	–

^a^Original patch indicates the patch in which the observed *L*.* delicatula* was fluorescent-marked at the onset of the experiment.

^b^One individual was confirmed.

^c^Minimum distance that *L*.* delicatula* adult could have traveled to achieve among-patch dispersal was estimated by measuring the shortest distance between two patches where adult was marked and marked adult was observed.

**Figure 4:**
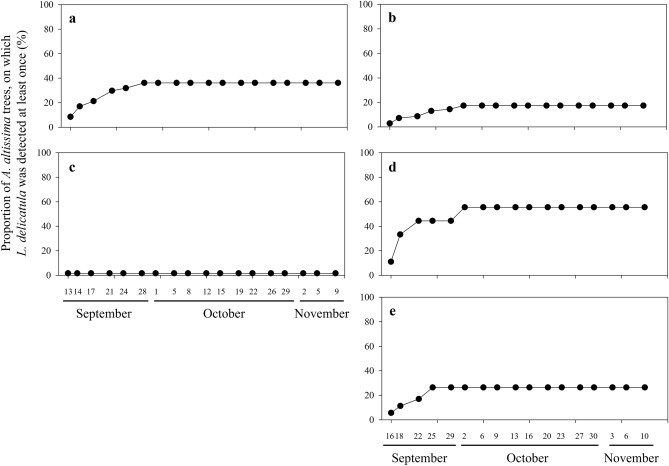
Proportion of *Ailanthus altissima* trees on which fluorescent-marked *Lycorma delicatula* was detected at least once during the observation period from September to November in patches C and D (**a** and **c**) from Tan stream and patches E–G (**b**, **d**, and **e**) from Gyeongan stream.

Fluorescent-marked SLF also displayed within-patch movement in most of the study patches during the observation period (Fig. 4). For within-patch movement, patches A and B were not included in analysis due to low numbers of *A*.* altissima* (< 5 trees) in the patches (Table 1). Except for patch C, the cumulative proportions of *A*.* altissima* trees on which marked SLF were detected gradually increased in September. In patch D of Tan stream, the number of trees with fluorescent-marked SLF detection gradually increased from the initial 9–36% by September 28th until the end of the survey (Fig. 4c). Similar to patch D, the proportion of *A*.* altissima* trees with fluorescent-marked SLF detected at least once gradually increased until October 2nd in Gyeongan stream. The overall number of trees increased gradually from the initial 3%, 11%, and 6% to 17%, 56%, and 26% in patches E, F, and G, respectively (Fig. 4).

### Oviposition pattern of SLF

**Figure 5:**
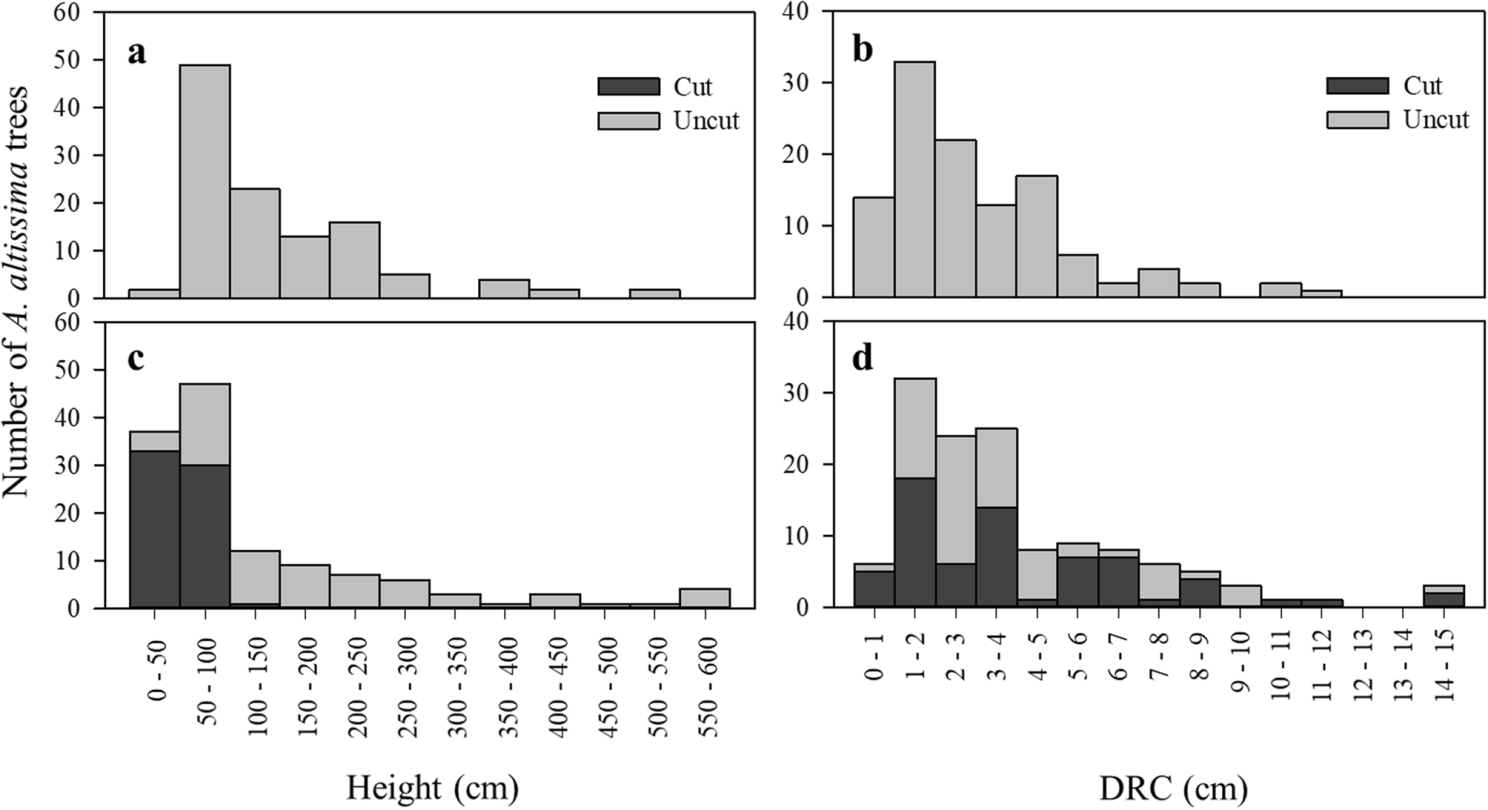
Frequency distributions of *Ailanthus altissima* tree height and diameter at root collar (DRC) in Tan stream (**a**, **b**) and Gyeongan stream (**c**, **d**).

In Tan stream, the tree heights of *A*.* altissima* ranged from 0.40 to 5.15 m, and in Gyeongan stream, the heights ranged from 0.20 to 5.90 m (Fig. 5a,b). Among the 116 trees surveyed in Tan stream, those < 2.5 m in their heights comprised 89%, and similarly, in Gyeongan stream, 86% of the 131 trees surveyed were < 2.5 m (Table 2; Fig. 5a,b). The DRC of *A*.* altissima* trees ranged from 0.32 to 11.15 cm and from 0.64 to 14.33 cm in Tan and Gyeongan streams, respectively (Fig. 5c,d). Trees with DRC < 5 cm were dominant comprising 85% and 72% of *A*.* altissima* trees in Tan stream and Gyeongan stream, respectively. Trees of which trunks were cut off by local administration were present only in Gyeongan stream comprising 45% of *A*.* altissima* trees in the stream.

**Table 2:** Characteristics of *Ailanthus altissima* tree and egg masses of *Lycorma delicatula* found in study patch.

Site	Patch	# of tree			Egg mass detected			# of egg in egg mass		Scattered eggs detected	
		Surveyed	Detected	Trunk cut off	< 2.5 m	> 2.5 m	Total	Total	Mean ± SE	#	# of eggs
Tan stream	A	4	0	0	0	0	0	–	–	0	0
	B	4	0	0	0	0	0	–	–	0	0
	C	61	8	0	14	2	16	410	29.29 ± 2.51	0	0
	D	47	12	0	53^a^	0	53	1792	33.81 ± 2.56	6	237
	Subtotal	116	20	0	67	2	69	2202	32.87 ± 2.10	6	237
Gyeongan stream	E	69	13	23	49	31	80	1804	37.58 ± 3.85	0	0
	F	9	2	0	4	0	4	122	30.50 ± 2.90	0	0
	G	53	3	36	6	0	6	129	21.50 ± 1.71	1	15
	Subtotal	131	18	59	59	31	90	2055	35.43 ± 3.26	1	15
Total		247	38	59	126	33	159	4257	34.06 ± 1.88	7	252

^a^In patch D, two egg masses without waxy layer were observed.

From the two streams, we found a total of 159 egg masses on 38 out of 247 *A*.* altissima* trees (Table 2). In Tan stream, 67 out of 69 egg masses were located < 2.5 m above the ground, whereas 59 out of 90 egg masses were detected < 2.5 m above the ground in Gyeongan stream. In both streams, ca. 30% of the egg masses were located < 20 cm above the ground. However, a substantial proportion of egg masses in Gyeongan stream were detected > 2.5 m above the ground, yielding 34% of the total egg masses (Table 2). 126 egg masses located < 2.5 m above the ground contained 4257 eggs, yielding an average of 34.06 ± 1.88 (Mean ± SE) eggs per egg mass (Table 2). In addition, we found 252 scattered eggs on seven *A*.* altissima* trees.

Using a generalized linear mixed model, the effects of tree height, DRC, and their interaction, and cut status on the number of egg masses on *A*.* altissima* trees in study patches were evaluated. Significant effects of tree height (*F*_1,241_ = 12.78, *P* < 0.001) and DRC (*F*_1,241_ = 12.54, *P* < 0.001) were detected (Supplementary information SI 1).

## Discussion and conclusion

To reliably track SLF adults, we tested the effect of fluorescent marking on survivorship and flight ability and found no significant adverse effects of fluorescent-marking on SLF adults. Furthermore, the durability of the method was confirmed in the field survey of this study, in which fluorescent-marked individuals were recaptured after a maximum of 47 days. Previously, Nixon et al.^23^ reported that fluorescent marking did not significantly affect horizontal and vertical dispersal capacity of SLF nymphs and adults. Moreover, Keller et al.^24^ observed dispersal of SLF nymphs in a deciduous forest, where fluorescent-marked individuals were detected a maximum of 65 m away from the release point. Therefore, the fluorescent marking system can be an effective tool to study the movement of SLF for both nymphs and adults. In addition to the conventional use of fluorescent marking for nighttime tracking^21,22,25,26^, fluorescent marking was effective in this study for the detection of SLF under the shadow created by tree canopy even during daytime.

As previously reported from many mark-recapture studies, the recapture rate of fluorescent-marked SLF also showed a consistent decrease during the study period with a rapid decrease during the early survey period^24,27,28^. Indeed, the recapture rates dropped on average to 10% during the first two weeks. Nevertheless, our results provide empirical evidence for the first time demonstrating active dispersal of natural SLF population among *A*.* altissima* patches. During the survey, we recorded 12 cases between September and October in which SLF individuals that had been marked from different patches at the onset of the experiment, were found in study patches. Based on these observations, we confirmed that at least seven SLF individuals displayed among-patch dispersal. Marked SLF were recaptured up to 27 days after the onset of the survey. In general, the marked individuals were found in *A*.* altissima* patches located close to the original patches from which SLF were marked at the onset of the survey. The minimum dispersal distance of marked SLF ranged between 10 and 60 m in most cases. Indeed, SLF has been reported to show descending flight behavior and short-range dispersals with each flight-bout ranging from 20 to 40 m in field conditions^29,30^. Interestingly, one marked SLF in this study was found to have dispersed at least 1.7 km within 22 days suggesting strong dispersal capacity during its oviposition period. Although it has not been evaluated in field conditions, Wolfin et al.^30^ suggested that the potential flight distance of SLF could be > 3 km based on laboratory observation.

Based on the survey of egg masses, we found that the tree height and DRC of *A*.* altissima* had significant effects on the number of egg masses on *A*.* altissima* trees. In addition, we observed significant positive correlations between these tree characteristics and the number of egg masses. These finding can serve as important information to prioritize sampling and management efforts based on tree characteristics especially when targeting the removal of egg masses from *A*.* altissima*. Indeed, a large-scale management practice was conducted in South Korea to identify and remove SLF egg masses during the early stages and outbreak of this invasive species^31^. With regard to vertical locations of egg masses on *A*.* altissima*, 79.2% of the masses were found within 2.5 m above the ground. In the surveyed areas, *A*.* altissima* trees were < 6 m in their height due to their age and local management practices such as tree trunk cutting. This would make it easier for investigators to locate and remove SLF egg masses for management practice. However, caution is needed when surveying substantially larger *A*.* altissima* as reported in previous studies in the US. Keller et al.^32^ found that 75% of egg masses were located above 6 m on *A*.* altissima* trees when the tree heights ranged from 5.5 to 23.8 m, and the lowest number of egg masses was located zero to 3 m above the ground.

In this study, we found that SLF actively dispersed in *A*.* altissima* patches during oviposition period and oviposited only on a small number of *A*.* altissima* trees within the patch. That is, although SLF adults were frequently observed on *A*.* altissima* trees while feeding or resting during the study, only a small portion of those trees were used for oviposition sites by SLF. This oviposition pattern of SLF may be attributed to the differential importance of *A*.* altissima* as feeding host versus oviposition substrate. SLF adults display higher preference towards *A*.* altissima* compared with other host plants from late August in South Korea and the US^13,18^. Nevertheless, SLF are known to deposit eggs on surfaces of various substrates including 28 taxa of host plants and inorganic materials such as stones and metal fences^12,20^. Indeed, a large number of SLF egg masses were detected on *E*.* danielli* and cement walls in the vicinity of *A*.* altissima* patches inspected in our survey (personal observation). Therefore, further studies are warranted to examine how SLF adults utilize different oviposition substrates following feeding on preferred host plants such as *A*.* altissima* and how this alternation would affect the likelihood of successful overwintering of egg masses or their survival against natural predators.

As one of the major host plants of SLF, *A*.* altissima* is already present in many countries expanding its geographical range, and the invasion of SLF is expected to be facilitated by the distribution of *A*.* altissima*. Especially in South Korea and the US, this invasive insect displays preference toward *A*.* altissima* in September through November when adults mate and oviposit, highlighting the importance of *A*.* altissima* in the life history of SLF^4,13,18^. In this study, we report for the first time dispersal patterns of natural SLF populations on *A*.* altissima* with a maximum of 1.7 km dispersal distance between *A*.* altissima* patches. We identified significant traits of *A*.* altissima* affecting the number of SLF egg masses on the trees. Our findings provide baseline information for developing proactive and efficient management strategies against SLF based on their dispersal and oviposition patterns on a major host plant.

## Materials and methods

### Fluorescent marking

Dispersal of SLF adults was tracked using a fluorescent marking system (FMS), which has been demonstrated to be applicable for multiple insect species including SLF nymphs^21,22,24^. To mark the SLF, either red, yellow, or blue fluorescent paint (#1166R, #1166Y, #1166B, BioQuip Products, USA) was diluted with distilled water (1:4). The mixture was then gently sprayed three times (ca. 20 mg each time) on each SLF individual using a mist sprayer from a distance of 30–50 cm (SI 2). Throughout the field survey, a handheld ultraviolet (UV) laser (PX 600 mW, class IIIB purple laser, 405 nm, Big Lasers, USA) was used to detect fluorescent-marked SLF individuals^25^.

### Effect of fluorescent marking on SLF

Prior to field survey, the potential effects of fluorescent marking on the survivorship and flight behavior of SLF adults (sex ratio 1:1) were evaluated. SLF adults were collected using sweeping nets (BioQuip Products, USA) from Gyeonggi-do, South Korea (37°47′85.95″ N, 127°11′64.58″E) in September 2020. Two hours after fluorescent marking of SLF, both fluorescent-marked and unmarked SLF were subjected to survivorship and flight behavior assessment.

Survivorship of insects was measured on two *A*.* altissima* trees (ca. 2 m in height) located in Gachon University, South Korea (37°45′38.50″N, 127°13′37.75″E). Two fluorescent-marked and two unmarked insects were placed in a cylindrical mesh cage [25 × 30 cm (radius × height)] enclosing a tree branch; a total of 20 groups were tested (n = 40). Then, survivorship of SLF was determined once every two days until no individuals were alive. Survivorship was compared between fluorescent-marked and unmarked SLF using Kaplan-Meir survivorship analysis (JMP 12, SAS Institute Inc., USA).

The effects of fluorescent marking on flight behavior were evaluated in an open space (986 m^2^) in Gachon University, South Korea (37°45′08.37″N, 127°12′79.69″E) at 26 ± 1 °C and a relative humidity of 30 ± 5%. To induce flight of SLF adults, a wooden square rod [3 × 3 × 100 cm (width × length × height)] was established upright at the center of the arena. The SLF adult was placed individually 10 cm away from the top on the wooden square rod. To minimize any unnecessary stimuli from experimenter, SLF flight was induced by following the same sequence: once the insect climbed up the rod and oriented itself staying still to a random direction, then an experimenter carefully positioned at the back of the insect and gently pecked the forewings using tweezers to initiate its flight^33,34^. Pecking was intended to mimic predatory behavior of birds. Once the insect jumped away, an operator followed the individual until it landed on the ground (n = 30). The experiment was conducted for 2 h between 13:00–15:00 and marked and unmarked SLF were randomly tested during the evaluation. The number of pecks to initiate the flight, flight duration, and flight distance of SLF were compared using *t-*test (JMP 12, SAS Institute Inc., USA).

### Field study sites

Dispersal patterns of SLF adults in *A*.* altissima* patches and their oviposition patterns were investigated in multiple *A*.* altissima* patches located along two streams in Gyeonggi-do, South Korea: Tan stream in Seongnam-si (37°48′01.80″N, 127°11′56.03″E) and Gyeongan stream in Gwangju-si (37°41′54.21″N, 127°27′12.37″E). Both Tan and Gyeongan streams run along suburban residential areas in their respective cities, with pedestrian lanes built along the streams. We selected seven *A*.* altissima* patches as study patches when more than 10 SLF adults were found per patch (Fig. 3). In the study patch, all SLF individuals or ca. up to 30 adults were florescent-marked. In addition, when the number of SLF adults was less than 10 from an *A*.* altissima* patch, those patches were designated as neighboring patches (Fig. 3). Dispersal and oviposition of SLF adults were monitored from both study and neighboring patches during the study.

In Tan stream, four study patches (patches A–D) and one neighboring patch, which were distributed over ca. 1760 m, were selected (Fig. 3a). Areas around the patches were generally covered with grass and shrubs, and the areas were occasionally managed by local administration. Deciduous trees were regularly planted along the pedestrian lanes. There were a total of four, four, 61, and 47 *A*.* altissima* trees in patches A to D, respectively (Table 2). Compared with Tan stream, *A*.* altissima* patches were located closely to each other in Gyeongan stream: three study patches (patches E–G) and three neighboring patches were spread over only ca. 90 m (Fig. 3b). Vegetation surrounding *A*.* altissima* patches consisted of grasses and small shrubs as well as deciduous trees planted along the border of residential area nearby. There were a total of 69, nine, and 53 *A*.* altissima* trees in patches E to G, respectively (Table 2). Unlike Tan stream, 45% of *A*.* altissima* trees had trunks having cut off by local administration in Gyeongan stream (Table 2; Fig. 5).

### Dispersal pattern of SLF on *A*.* altissima*

Three fluorescent paint colors were used to mark SLF individuals in the study patches (Fig. 3; SI 2). Insects that took off during marking were captured and excluded from the experiment. Among the selected study patches, SLF adults were generally distributed throughout each patch, while SLF adults were observed only from one out of 61 *A*.* altissima* trees in patch C. As a result, in Tan stream, 15 (color of paint used to fluorescent-marking; red), 31 (yellow), 11 (blue), and 32 (red) adults were marked from patches A to D, respectively, whereas in Gyeongan stream, 30 (red), 30 (blue), and 33 (yellow) adults were marked from patches E to G, respectively. Starting from September 14th, 2020 in Tan stream and September 18th in Gyeongan stream, fluorescent-marked SLF adults on *A*.* altissima* trees in both study and neighboring patches were counted with a UV laser twice a week (Fig. 3). Survey continued until no individuals were observed from the study patches.

### Oviposition pattern of SLF on *A*.* altissima*

Oviposition pattern of SLF was surveyed on all *A*.* altissima* trees in the study patches in December in both streams (Table 2). For the survey, SLF egg masses were categorized into three types as follows: egg mass with waxy layer, egg mass without waxy layer, and scattered eggs (SI 3). Eggs that were not covered with waxy layer and did not form aggregates were categorized as scattered (SI 3). In the field, *A*.* altissima* trees were visually inspected to identify SLF egg mass, and the number of egg masses and their distances from the ground were recorded. In addition, the number of eggs per egg mass was recorded for egg masses located < 2.5 m above the ground. When egg masses were covered with waxy layer, brushes were used to gently remove the powder-like waxy layer.

To characterize the *A*.* altissima* trees surveyed for SLF egg mass, we recorded the following morphological characteristics: tree height, DRC, and cutting status. Heights of *A*.* altissima* trees < 2 m were measured using an iron steel measuring tape, and those > 2 m were estimated with reference to the investigator’s height. DRC of *A*.* altissima* was determined by measuring the circumference of the stem at the root collar using a tape measure. In case of multi-stemmed *A*.* altissima* trees, the diameter of each stem was first determined individually, and the DRC of the tree was calculated using the following equation:

$$ DRC = \sqrt {\mathop \sum \limits_{i = 1}^{n} d_{i}^{2} } $$

where *n* is the number of stems and *d*_*i*_ denotes the stem diameter of the *A*.* altissima* tree.

The effects of *A*.* altissima* height, DRC, their interactions, and cutting status of the trees on the number of egg masses were analyzed using a generalized linear mixed model (GLMM) assuming a Poisson distribution with a log link function (JMP 12, SAS Institute Inc., USA). Patch was included as random effect in the model. Due to concerns over collinearity between tree height and DRC, variance inflation factor (VIF) was calculated: VIF > 5 generally indicates collinearity^35,36^. VIF between height and DRC was 1.56, and therefore the two variables were included together in the GLMM model.

### Policy statement

Experiments involving *Ailanthus altissima* were conducted in compliance with relevant institutional, national, and international guidelines and legislation.

## Supplementary Information

Supplementary Information.
