# Supplementary material for: Dispersal and oviposition patterns of Lycorma delicatula (Hemiptera: Fulgoridae) during the oviposition period in Ailanthus altissima (Simaroubaceae)
Source: Sci Rep. 2022 Jun 15;12:9972. doi: 10.1038/s41598-022-14264-0 (PMC9200975; doi:10.1038/s41598-022-14264-0)
Supplement: Supplementary file 1 — Supplementary Information. [file 41598_2022_14264_MOESM1_ESM.docx]

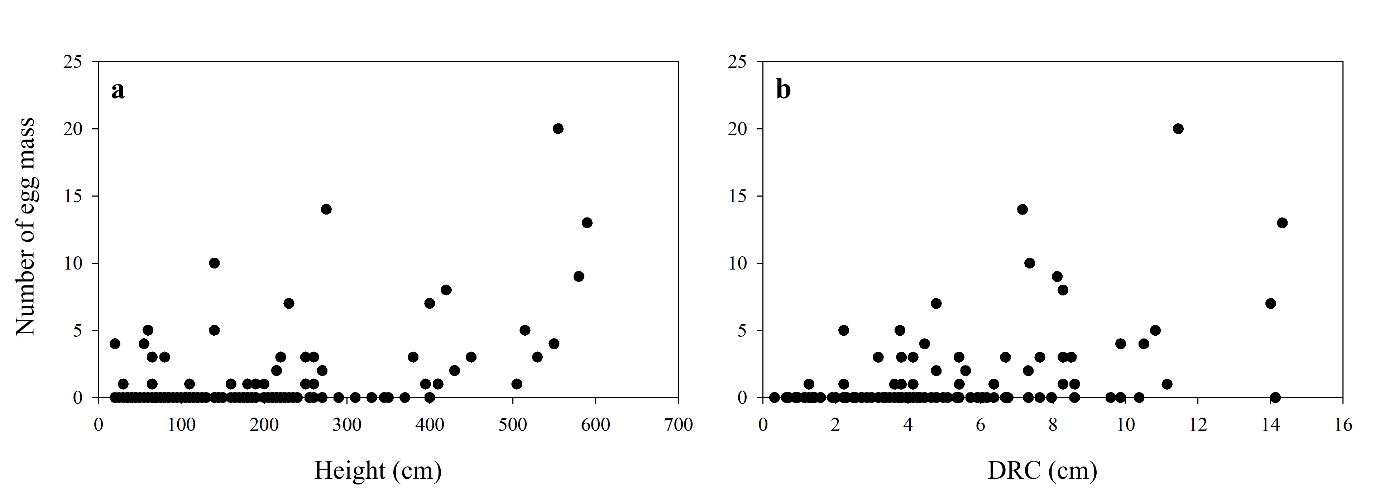


Supplementary information SI 1. Scatterplot of the number of egg mass of *Lycorma delicatula* and over **a** tree height and **b** diameter root collar (DRC) of *Ailanthus altissima* trees.


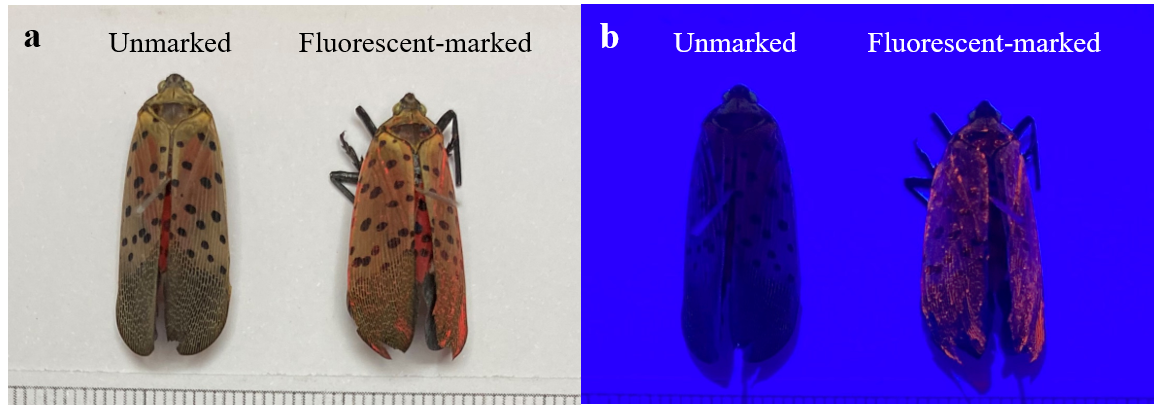


SI 2. Unmarked and fluorescent-marked *Lycorma delicatula* adults under **a** white light and **b** ultraviolet (UV) light illuminated in the dark.


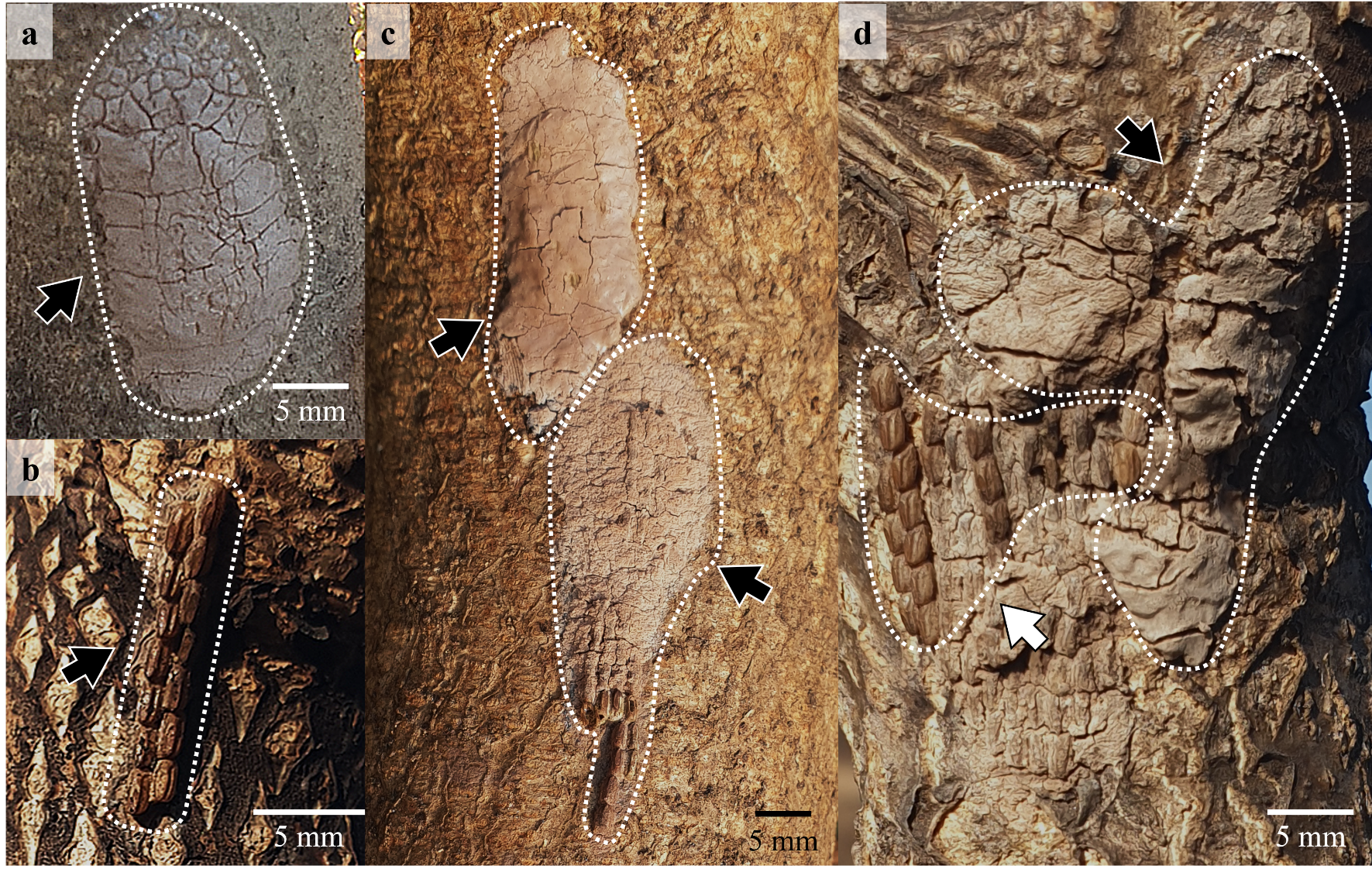


SI 3. Different types of egg masses or eggs of *Lycorma delicatula* found on *Ailanthus altissima* trees. **a** Egg mass with waxy layer. **b** Egg mass without waxy layer. **c** Two separate egg masses covered with waxy layer. **d** A single egg mass covered with waxy layer and adjacent scattered eggs. Black arrows indicate egg masses with or without waxy layers, and the white arrow indicates scattered eggs.
